# Investigation of Incidents and Trends of Antimicrobial Resistance in Foodborne Pathogens in Eight Countries from Historical Sample Data

**DOI:** 10.3390/ijerph17020472

**Published:** 2020-01-10

**Authors:** Katherine Yang, Annie Wang, Matthew Fu, Aaron Wang, Kevin Chen, Qian Jia, Zuyi Huang

**Affiliations:** 1 Department of Chemical Engineering, Villanova University, Villanova, PA 19085, USA; katherineyang2002@gmail.com (K.Y.); agani.first@gmail.com (A.W.); zmfu2002@gmail.com (M.F.); aaronwangca@gmail.com (A.W.); maykevin.chen@gmail.com (K.C.); 2 Department of Health, Nutrition & Exercise Sciences, Immaculata University, Immaculata, PA 19345, USA; qjia@immaculata.edu

**Keywords:** foodborne pathogens, antimicrobial resistance, principal component analysis, hierarchical clustering

## Abstract

Antimicrobial resistance (AMR) causes millions of illnesses every year, threatening the success of lifesaving antibiotic therapy and, thus, public health. To examine the rise and spread of antimicrobial resistance around the world, our study performs a multivariate statistical analysis of antimicrobial resistance gene data from eight different countries: the US, the UK, China, Brazil, Mexico, Canada, Australia, and South Africa. Multi-dimensional data points were projected onto a two-dimensional plane using principal component analysis and organized into a dendrogram utilizing hierarchical clustering to identify significant AMR genes and pathogens. Outlier genes/pathogens were typically involved in high occurrences of antimicrobial resistance, and they were able to indicate the trend of antimicrobial resistance in the future. Statistical analysis of the data identified: (1) *tet(A)*, *aph(3″)-Ib*, *aph(6)-Id*, *blaEC*, *blaTEM-1*, *qacEdelta1*, *sul1*, *sul2*, and *aadA1* as the nine most common AMR genes among the studied countries; (2) *Salmonella enterica* and *E. coli* and *Shigella* as the most common AMR foodborne pathogens; and (3) chicken as the most prevalent meat carrier of antimicrobial resistance. Our study shows that the overall number of reported antimicrobial resistance cases in foodborne pathogens is generally rising. One potential contributing factor for this is the increasing antimicrobial usage in the growing livestock industry.

## 1. Introduction

Antimicrobial resistance is increasingly a threat to global public health [1]. AMR-related infections limit the efficacy of lifesaving antibiotic therapy necessary for the treatment of infectious disease and limit the success of advanced surgical procedures like organ transplants [2,3]. This results in millions of illnesses and deaths worldwide. For example, the US Centers for Disease Control and Prevention (CDC) estimated that AMR bacteria account for over 2 million illnesses and at least 23,000 deaths in the US every year [4]. Similarly, it is estimated that 426,000 infections and 33,000 deaths from antimicrobial resistant microorganisms occur in the European Union every year [5]. Furthermore, AMR places a considerable medical and financial burden on global societies and health care systems as a whole [1,3,6,7,8]. It is therefore essential to develop a better understanding of the cause and spread of AMR around the world.

As a result of selection pressures provided by the usage of antimicrobials, resistance genes utilize various mechanisms to inhibit the antimicrobial’s effects—enzymatic inactivation, antimicrobial target modification or protection, drug permeability reduction, and drug efflux [2]. These genes are becoming increasingly prevalent. In response to the need for further research in the spread of resistant foodborne pathogens and illness, the NCBI Pathogen Detection Isolates Browser (NPDIB) was created to integrate bacterial pathogen genomic sequences originating in food, patients, and environmental sources [9]. It analyzes samples from around the world and compares their genomic sequences to others already in the database. When AMR foodborne pathogens access humans, the AMR genes may be passed from the pathogens to human cells via horizontal gene transfer (HGT) through mechanisms of transformation, transduction, or conjugation [10]. This is implied by a study in which 13,514 high confidence HGT genes had been found in 308 human microbes [11]. The development of antibiotic resistance in foodborne bacteria and in HGT can significantly endanger human health. NPDIB clusters and identifies related sequences to uncover potential food contamination outbreaks and resistance genes. Through the database, people can search for foodborne pathogen isolates and identify pathogens with particular resistance genes. In previous literature, the database was typically only used to compare the DNA from detected pathogens to those in the database or provide data for a historical analysis of resistance in specific pathogens [12,13]. Little research, however, has been done to examine specific AMR genes over large geographic areas.

Currently, only one previous study used the database to study specific resistance genes [14]. That 2019 study examined the spread of resistance genes across six different US states. It found common antimicrobial-resistance genes in both geographically near and geographically far states in the US. However, it did not study the historical occurrence trends of either antimicrobial resistance genes or pathogens. Therefore, the study was unable to indicate how the antimicrobial resistance issue evolved over time. In order to address these gaps, our study aims to expand the previous research by extending the scope of its work and identifying genes with increasing occurrence over time. A multivariate statistical method like principal component analysis [15,16] was used in this work to visualize the data into comparable points on a two-dimensional plane. On the basis of this, the hierarchal clustering approach [17,18,19] was further used to identify the genes, foodborne pathogens, and meats that were mostly involved in antimicrobial resistance. The occurrence trends of specific resistant genes and foodborne pathogens over time were examined over eight different countries in geographically different areas around the world—the US, the UK, China, Brazil, Mexico, Canada, Australia, and South Africa—which have the most amount sample data in NPDIB for different continents.

## 2. Materials and Methods

### 2.1. Data Source: NCBI Pathogen Isolates Browsers

**Figure 1:**
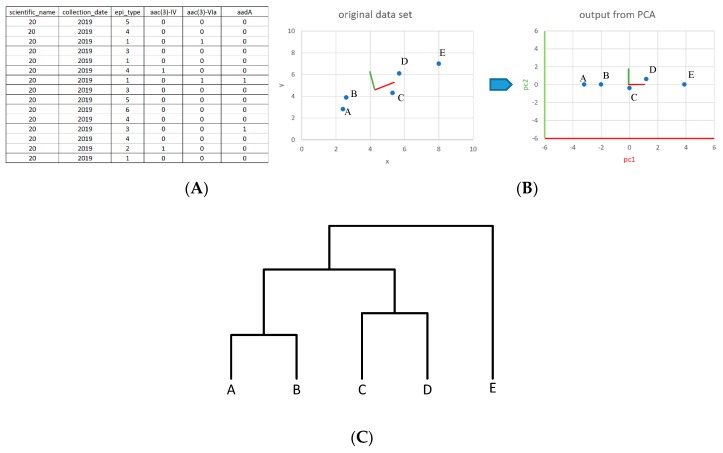
The general procedure of multivariate statistical analysis implemented in this study: (**A**) A portion of the data matrix extracted from NCBI Pathogen Detection Isolates Browser Database for the US; (**B**) principal component analysis was used to reduce the data matrix dimensions so that the data points (e.g., genes/pathogens/meats) can be visualized in a two dimensional space (e.g., the *x*-*y* space can be reduced to the PC1 space); (**C**) the hierarchical clustering approach was used to separate the data points on the basis of a two-dimensional space produced by principal component analysis. Simple diagrams are used in (**A**–**C**) to illustrate the general steps for principal component analysis and hierarchical clustering.

The NPDIB database was created to monitor patterns and trends of illness-causing pathogens and genes. These pathogens were sampled from food, the environment, and human patients by health agencies in various countries, such as the Centers for Disease Control and Prevention, the U.S. Food and Drug Administration, the U.S. Department of Agriculture, and Public Health England. The database contains information on geographic location, sample time, isolation type, food source, and host. In this study, data for hundreds of samples collected from 2010 to 2019 were extracted from the NCBI database for eight countries: the US, the UK, South Africa, China, Australia, Canada, Mexico, and Brazil. These countries have the most data in the NPDIB database for the continents where they are located, making them the most suitable countries for analysis. In addition, the countries were selected because of their geographical, cultural, and developmental diversity. The chosen countries include highly developed countries, such as the US and the UK, along with less industrialized countries like South Africa and Brazil. Some countries have very high populations while others have fewer people. Using MATLAB software programs, the data for each country was organized into matrices in which each row represented one sample and each column represented one of five categories of information: (1) the scientific name of the foodborne pathogen, (2) the collection date, (3) the sample source (e.g., turkey or chicken), (4) the number of AMR genes identified in the sample, and (5) individual AMR genes (with a value of zero/one that indicated the absence/presence of the corresponding gene in the sample). Figure 1 shows the general procedure of the multivariate statistical analysis implemented in this work. Figure 1A,B illustrate the steps of extracting data from the NPDIB database to the matrix format for further statistical analysis.

### 2.2. Statistical Analysis

Given each sample’s large number of dimensions, principal component analysis (PCA) was applied to each country’s data to visualize the complex data matrices into two-dimensional plots, providing a clear image of data and their relationships. Utilizing dimensionality reduction, PCA created new individual dimensions that can retain the most information in the data matrix. These new dimensional variables, called principal components, were orthogonal vectors that represented different levels of variance in the data. In order to offer a strong representation of the data, a sizable amount of variation needed to be accounted for by the principal components used in the visual illustration of the data. By looking at the proportion of variance explained (PVE), PC1 (the vector with the greatest PVE and, thus, the highest variance in its projections), and PC2 (the vector with the second highest PVE and variance in its projections) were chosen to create the two dimensional graph of the data. When plotting the sample data points, PC1 was used as the *x*-axis and PC2 as the *y*-axis. The graph created revealed clusters of similar data points along with significant outliers (Figure 1C).

On the basis of the PCA plots, hierarchical clustering was used to separate the studied objects (e.g., AMR genes, foodborne pathogens, and meats) so that the objects showing similar involvement with antimicrobial resistance were put into the same group. Initially treating each observation as a unique object, hierarchical clustering merged the two objects that were in the closest proximity to each other and, thus, had the highest similarity to form a new cluster. It then continued to merge close clusters until only one remained. The hierarchical clustering output a dendrogram, a tree diagram that represents different clusters and how they merge together. The vertical height of the link between two objects (i.e., foodborne pathogens, AMR genes, or meats) revealed their proximity and similarity, in that the smaller the height at which the two objects were merged, the more similar their involvement in antimicrobial resistance. In Figure 1C, for example, Genes E and F were the closest in proximity in the PCA plot and were detected in similar samples. They, thus, had the lowest linkage in the dendrogram. The dendrograms produced by the antimicrobial resistance data were analyzed, and significant outlier genes and foodborne pathogens were identified. Those outlier genes and foodborne pathogens were further confirmed from the PCA plot. Special attention should be paid to their indications of antimicrobial resistance for the following reasons: (1) they were of high occurring frequencies in the isolate samples; (2) the genes they indicated were involved in the resistance to various antimicrobials; (3) the pathogens they indicated carried various AMR genes. These genes and foodborne pathogens were used to generate the historical occurrence profiles of frequently occurring genes and foodborne pathogens over time. The profiles of absolute frequency were plotted for both AMR genes and pathogens to indicate the number of samples containing the selected gene or pathogen that was collected each year. In addition, the profiles of the relative frequency, which was calculated by the number of samples for a gene/pathogen in each year divided by the total number of samples obtained in that year, were also provided in this work so as to reduce bias resulting from the variation of sample numbers across different years. These graphs were then used to quantify the movement of AMR (upwards or downwards) by country based on the historical data from 2010 to 2019.

R, a programming language capable of performing statistical analysis and generating graphs [20], was used to create the analytical data set and perform the statistical analysis in this project. R offers many statistical and graphical packages and easily produces plots and calculations on arrays. For these reasons, it was well-suited for multivariate statistical analysis in this work.

## 3. Results

Data from the NPDIB was analyzed using PCA and hierarchical clustering in this section to identify: (1) the common highly-occurring antimicrobial-resistance genes in the studied countries: the US, the UK, South Africa, China, Australia, Canada, and Mexico; (2) the common highly-occurring foodborne pathogens carrying antimicrobial-resistance genes in each of the countries studied; (3) and the parallelism in antimicrobial resistance trends among the eight countries; (4) the similarities in the occurrence of AMR genes and foodborne pathogens in the selected eight countries.

### 3.1. Identification of Common Highly-Occurring AMR Genes in the Studied Countries

#### 3.1.1. Common Frequently-Occurring Antimicrobial-Resistance Genes for Individual Countries for All Years

**Figure 2:**
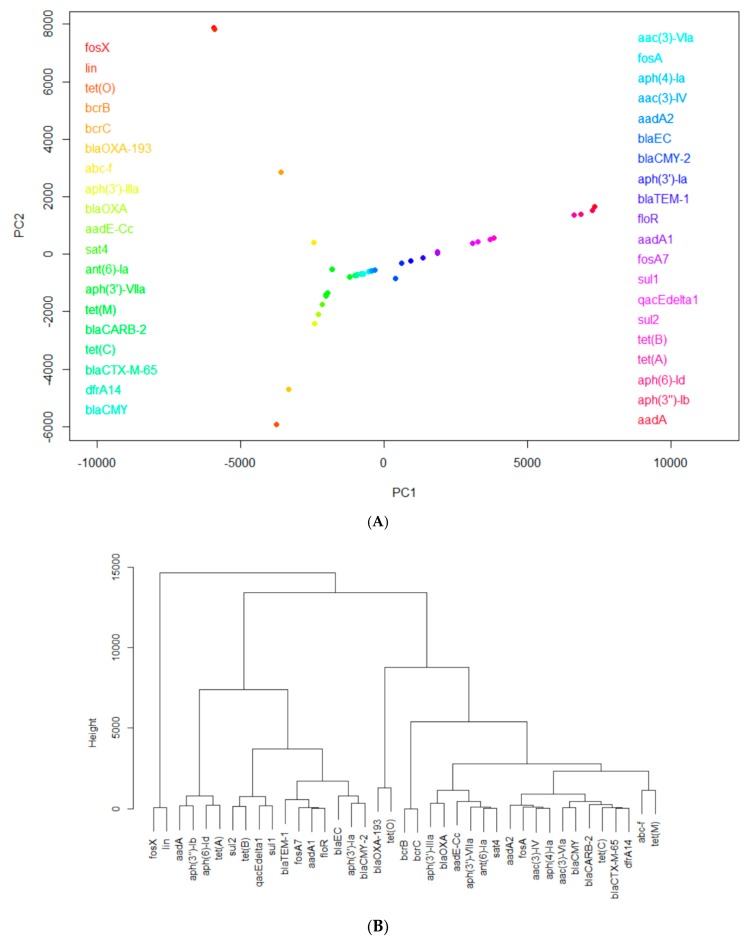
An illustrative example of PCA and hierarchical analysis: (**A**) projections of antimicrobial genes onto the PC1~PC2 space based on the occurrence of these genes detected in foodborne pathogens; (**B**) clustering of the genes based on their projections in (**A**).

Highly-occurring AMR genes in each of the eight countries were identified using PCA and hierarchical clustering. For each country, the antimicrobial resistance gene data for different pathogens was organized into a matrix, each row representing one gene and each column representing one pathogen. The elements in the matrix recorded the number of samples in which the gene in the row was detected in the pathogen shown in the column. As an example, Figure 2 shows the results from such analysis for the United States’ Data: Figure 2A projects the AMR genes onto the PC1~PC2 space and Figure 2B shows the hierarchical clustering constructed from the PCA projection. Genes in close proximity to each other in the principal component analysis plots had similar trends of occurrence and, thus, were grouped closely in the hierarchical clusters. As seen in the center of Figure 2A, the majority of genes—including *tet(C)*, *blaCMY*, and *fosA*—were grouped together in the principal component analysis, and were correspondingly clustered in the same branch of the hierarchical cluster, highlighting their similar trends of occurrence. However, genes with unique trends of higher levels of occurrence were separated from the majority of cluster of genes in PCA. For example, *fosX* and *lin* stand out from the rest of the genes, revealing their significance. Through detailed hierarchical clustering, Figure 2B illustrates this significance with a clear visual: it groups *fosX* and *lin*, along with the other highly occurring genes, into their respective clusters.

The AMR genes that stand out as the most frequently-occurring from the analysis of each country’s data are listed in Table 1. In Table 1, the genes are sorted by their frequency, and it can be seen that (1) *tet(A)*, *aph(3″)-Ib*, *aph(6)-Id*, *blaEC*, *blaTEM-1*, *qacEdelta1*, *sul1*, *sul2*, and *aadA1* are the nine most common AMR genes in the majority of the studied countries; (2) South Africa’s set of highly-occurring AMR genes are relatively unique; and (3) China harbors the greatest number of genes uncommon to the other seven countries. Supplementary Table S1 shows the mechanisms of the antimicrobial resistance associated with those frequently-occurring genes listed in Table 1.

#### 3.1.2. Historical Profiles of Highly-Occurring AMR Gene Occurrence from 2010 to 2019

One of the crucial aspects of the NPDIB data was the relative frequency of occurrence. Since there are a large number of AMR genes detected in each country, this section only focuses on the highly-occurring AMR genes that are listed in Table 1. The historical graphs of these genes should reflect the occurrence trend of the AMR genes in those countries. Genes that increased in occurrence frequency were identified and listed in Table 2 for all the eight countries studied.

### 3.2. Identification of Common, Highly Occurring Pathogens in the Studied Countries 

As in the previous section, the antimicrobial resistant gene data for each country was organized into a matrix. To isolate highly occurring antimicrobial resistant pathogens, the data was reorganized so that each row represented one pathogen and each column represented one gene. PCA plots and hierarchical clusters were then created for each country grouping pathogens with similar trends of resistance.

#### 3.2.1. Common, Highly Occurring Pathogens of Individual Countries for All Years 

**Figure 3:**
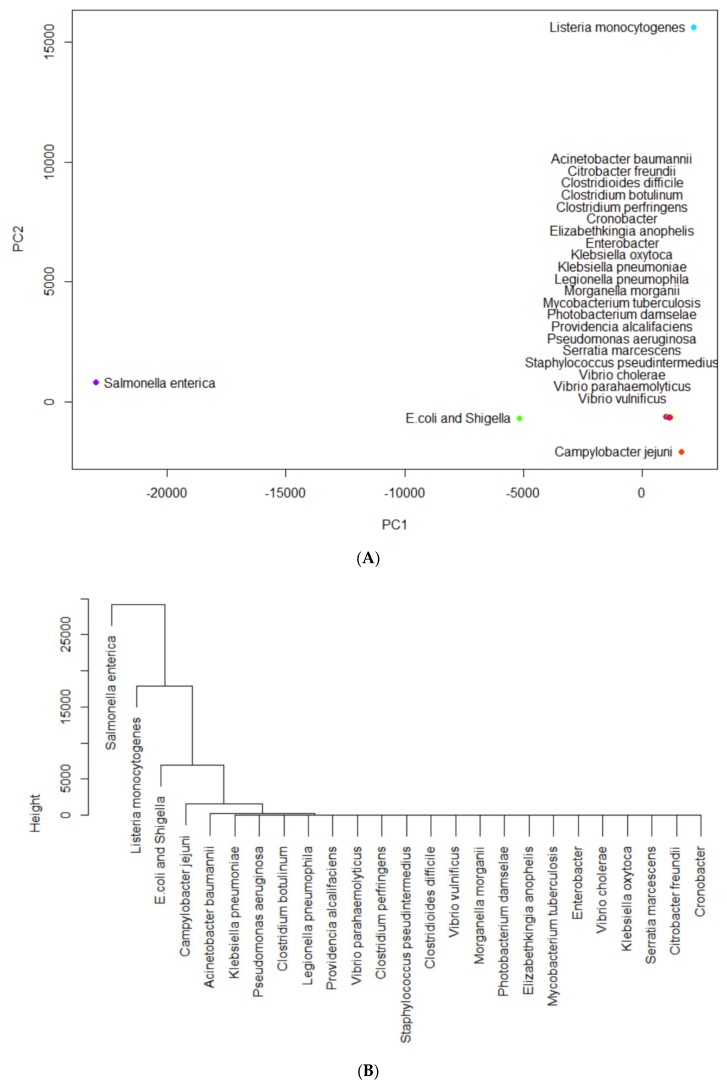
An illustrative example of PCA and hierarchical clustering: (**A**) projections of pathogens onto the PC1-PC2 space based on the resistance of antimicrobials; (**B**) clustering the pathogens based on their projections in (**A**).

The key highly occurring pathogens in each of the eight countries were identified using PCA and hierarchical clustering. As an example, Figure 3 shows the results from such analysis for the United States’ Data: Figure 3A projects the pathogens onto the PC1~PC2 space and Figure 3B shows the hierarchical clustering constructed from the PCA projection. As seen in Figure 3A, pathogens such as *Listeria monocytogenes* and *Salmonella enterica* stand out from the rest of the pathogens, revealing their notably high levels of occurrence. Figure 3B, through a detailed hierarchical clustering, illustrates this significance through a clear visual. It groups *Listeria monocytogenes* and *Salmonella enterica*, along with the other highly-occurring pathogens, into their respective clusters.

Through this process, it can be determined that the most highly-occurring pathogens in the US are: *Listeria monocytogenes*, *Salmonella enterica*, *E. coli* and *Shigella*, and *Campylobacter jejuni*. Each of these pathogens forms its own cluster alone and is extremely variant from the rest of the pathogens which form one big cluster apart from these four.

All the other countries were analyzed with PCA and hierarchical clustering in a manner similar to the analysis of the US. Highest occurring AMR pathogens are sorted and color-coded by their presence in each country (the US, the UK, South Africa, China, Australia, Canada, Mexico, and Brazil) in Table 3. *Salmonella enterica* and *E. coli* and *Shigella* were the most common highly-occurring pathogens. *Listeria monocytogenes* and *Klebsiella pneumoniae* were each highly-occurring in at least two of the countries studied, suggesting similarities and relations between specific partner countries. Similarly, as in the case for genes, China has the most unique AMR pathogens among the studied countries, *Enterobacter* and *Cronobacter*.

#### 3.2.2. Historical Profiles of Highly-Occurring Pathogen Occurrence between 2010 and 2018

**Figure 4:**
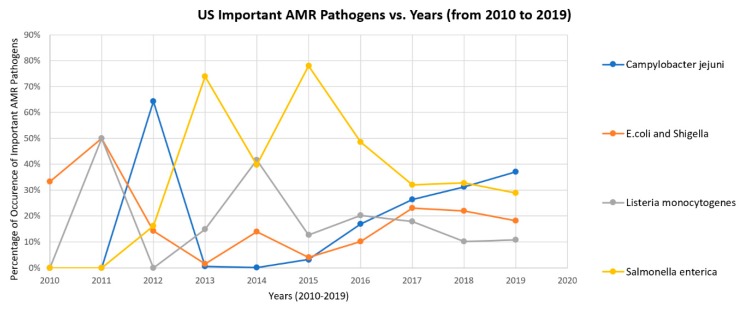
Frequency of highly-occurring pathogens over the years 2010–2019 in the US.

The relative frequency graphs of highly-occurring foodborne pathogens present in the US were plotted together in Figure 4 for an example. It can be seen that the occurrence of *Listeria monocytogenes* and *Salmonella enterica* generally decreased in recent years in the US. The occurrence of *Campylobacter jejuni* decreased first, but then increased since 2014. The historical profile of *E. coli* and *Shigella* looks similar to the one of *Campylobacter jejuni* but with a generally decreasing trend in 2018 and 2019. The occurrence trend of *E. coli* and *Shigella* is thus not regarded as an increasing one. A similar approach was implemented to the other selected countries to find the pathogens with a generally increasing occurrence. The result is shown in Table 4 where most countries have at least a foodborne pathogen increasingly detected in the samples. This implies that certain foodborne pathogens will keep carrying those AMR genes over time and that there is still an urgent need to combat antimicrobial resistance issues throughout the world.

### 3.3. Major Meat Carriers Found in the Studied Countries

**Figure 5:**
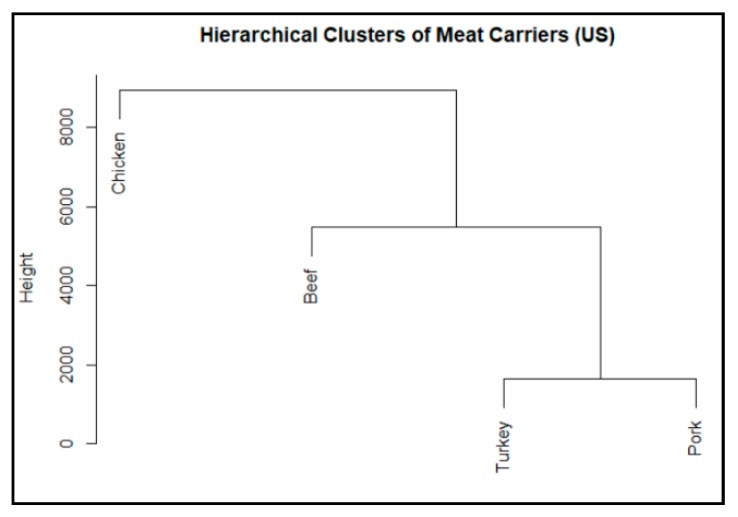
Hierarchical clusters of meat carriers are displayed for the US.

All the four major meats (beef, chicken, pork, and turkey) were studied through PCA and hierarchical clustering analysis of the AMR genes they carried for each of the countries studied. The hierarchical cluster diagrams for the US are provided in Figure 5 as an example. This diagram was used to rank the meat carriers in terms of their relative occurrence in carrying AMR genes in the range of 1 to 4, 1 represents the most and 4 represents the least relative occurrence. For example, in the US, chicken was ranked as 1, beef as 2, and turkey and pork were ranked equally as 4. The individual ranking for each country and the total ranking for all the studied countries are provided in Table 5.

From Table 5, a couple of trends can be identified: (1) overall rank of prevalent carriers are chicken, beef, pork, and turkey (with chicken as the most prevalent), which correlates well with the world per capita consumption of the different meats [21] and (2) beef and chicken are dominant in the US, Canada, and Mexico (three north American countries); pork and chicken are dominant in the UK, China, and Brazil; and beef is dominant in Australia with the other three meats being equal.

### 3.4. Identification of Similarity in Antimicrobial Resistance Occurrence in Studied Countries

**Figure 6:**
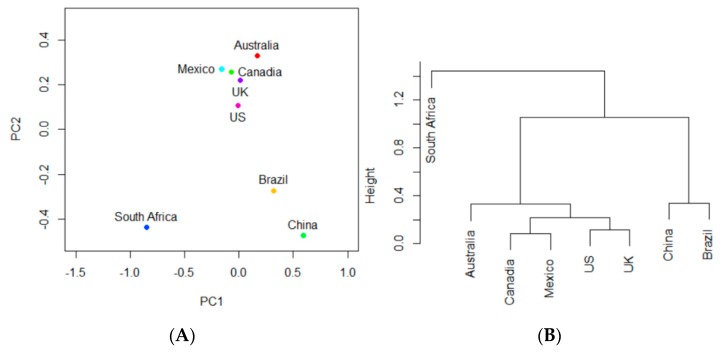
(**A**) Projections of South Africa, Mexico, Canada, the UK, the US, Brazil, China, and Australia onto the PC1-PC2 space based on the occurrence of AMR genes shown in Table 1; (**B**) clustering the countries based on their projections in (**A**).

Analysis was performed on the data to study the similarity in antimicrobial resistance in the eight selected countries. The data was first organized into a matrix in which each row represented one country and each column represented each highly-occurring AMR gene from Table 1. PCA (Figure 6A) and hierarchical clustering (Figure 6B) revealed the similarities in highly-occurring AMR gene frequencies and occurrences in the eight countries. In hierarchical clustering, countries connected together at a lower vertical level exhibited a greater similarity in the occurrences of AMR genes. Therefore, a few clusters are notable in Figure 6B: (1) the US and the UK, the most developed countries, are clustered together; (2) China and Brazil, high developing countries, are clustered together; (3) Canada and Mexico’s cluster is vertically very close to the US and the UK; and (4) South Africa is the most vertically unique and varying country.

**Figure 7:**
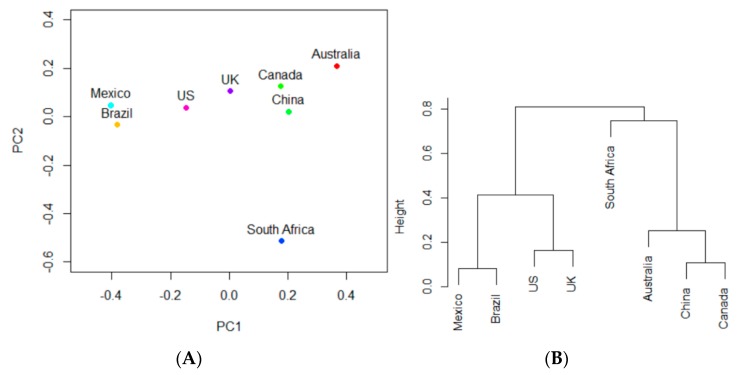
(**A**) projections of South Africa, Mexico, Canada, the UK, the US, Brazil, China, and Australia onto the PC1-PC2 space based on the occurrence of foodborne pathogens shown in Table 3; (**B**) clustering the countries based on their projections in (**A**).

Furthermore, PCA and hierarchical clustering analysis highlighted the similarity of the highly-occurring resistant pathogen occurrence across the eight countries studied. Figure 7A shows the projections of countries onto the PC1–PC2 space based on the occurrence of the foodborne pathogens shown in Table 3. Figure 7B displays the clustering of the countries based on their projections in Figure 7A. South Africa is again the most vertically unique, as its height in the hierarchical cluster is significantly higher than those of the other seven countries. It therefore exhibits the most unique and different patterns of resistance in its pathogens. Among the other seven countries, Brazil and Mexico were coupled together, and Canada and China were clustered together. As found previously, the US and UK also formed a clustered pair. Australia was located close to Canada and China, but was not in a cluster pair. Figure 7 is different from Figure 6, as the AMR genes can be carried by multiple pathogens. In other words, AMR genes and specific pathogens were not necessarily correlated in their occurrence. For example, the AMR genes can be carried by the pathogens not shown in Table 3, on the basis of which Figure 7 was generated.

## 4. Discussion

### 4.1. Absolute Occurrence versus the Relative Occurrence for Studying the Time Trend of Antimicrobial Resistance

**Figure 8:**
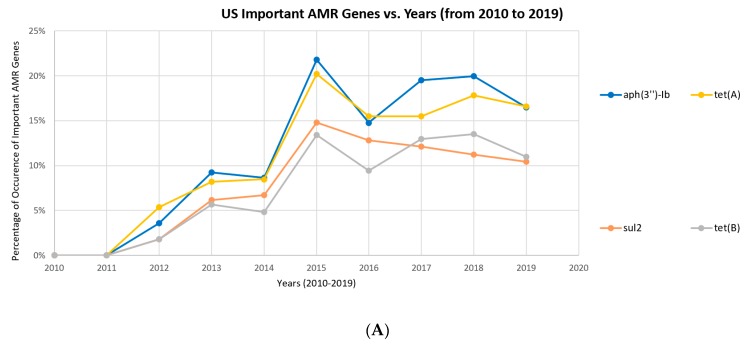

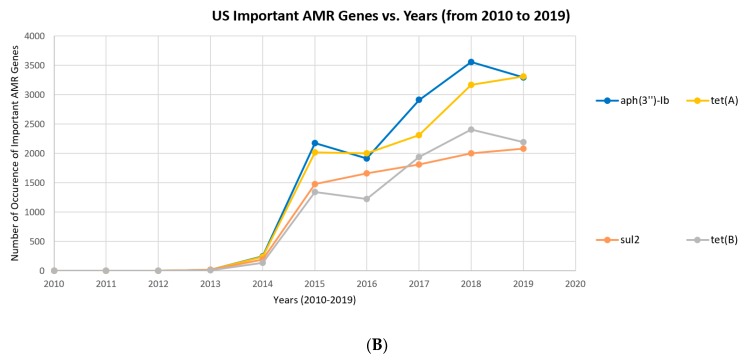
Frequencies of occurrence among the important AMR genes *aph(3″)-lb*, *tet*(*A*), *sul2*, and *tet*(*B*) between 2010 and 2019 in the US with (**A**) normalized and (**B**) absolute numbers of samples that contain the AMR genes.

Figure 8 shows the occurrence profiles of several representative genes occurring with increasing frequency. The relative occurrence, instead of the absolute occurrence, was preferred for studying the trend of antimicrobial resistance. This is because the number of available samples in early years (e.g., from 2010 to 2013) is relatively low compared to later years. The rise magnitudes shown in the absolute frequency profiles in Figure 8B are much higher than those shown in Figure 8A, even with only seven months of data available for 2019. Figure 8B indicates a strong increase in the absolute frequencies of AMR genes over time. However, this may be due to the fact that fewer samples were collected in the early years because of the limited interest from researchers and because fewer facilities were available for sample collection. The modern understanding of the rise of AMR may be inflated by the skewed absolute occurrences of resistance. However, the number of AMR genes showing a general increase in their relative occurrences from 2011 still implies the existence of a potential rise in the relative occurrence of AMR genes, regardless of the influence of the increased frequency of measurement.

### 4.2. Antimicrobial Usage and the Rising Overall Trend of AMR Genes

Figure 8 indicates an absolute frequency profile that shows a stronger increasing trend than the relative one from 2010 to 2019. In the eight studied countries, 43 different antimicrobial resistant genes were deemed highly-occurring. This upward trend shown in the absolute frequency profile occurs because of the following potential factors: (1) the increase in reports of antimicrobial resistance isolates because of developments in microbial measurement technology; (2) the population increase in those countries; (3) the increased populations with lower immune systems (e.g., people with obesity or nosocomial infections); and (4) the growth of antimicrobial usage in the livestock industry.

In particular, many countries are globalizing, joining the international meat trade web. Propelled by the rapid rise in participating countries, the meat industry is expected to grow to $1.5 T by 2022. This amount doubles the number from 2016, $714 B. The expansion of the livestock industry has led to a greater exchange of animal products—and, thus, their AMR genes and resistant pathogens—and an increased administration of excessive antibiotics in livestock. The rise in specific antimicrobial resistant genes in various countries may be correlated with the excessive administration of antibiotics to livestock. Such a relationship is most strongly seen in the UK, in which nearly all resistant genes were resistant to either aminoglycoside antibiotics, beta-lactam related antibiotics (cephalosporin, penam), sulfonamide antibiotics, or tetracycline antibiotics. Interestingly, this corresponds with the top four antibiotics used in livestock in the UK. While the use of antibiotics as growth promoters was banned in the UK in the early 2000s, antibiotics were still used in other sectors of the livestock industry, such as for prophylaxis. Of the antibiotics used on livestock in the UK, 37% was tetracycline, 26% penicillin (which utilizes beta lactam resistance), 11% sulfonamide, and 8% aminoglycosides [22]. These four antibiotics add up to 82% of UK antibiotic usage in livestock, implying a potential correlation between antibiotic usage in livestock and the rising trend of AMR genes [22]. On the other hand, the relative frequency profile (Figure 8A) does not show as strong an increasing trend as the absolute frequency profile (Figure 8B). This indicates that the aforementioned factors other than the growth of antimicrobial usage may be contributing to the increase in the absolute frequency profile.

Of the 43 highly-occurring antimicrobial resistant genes, only one had significant occurrences in all 8 of the studied countries—*tet(A).* As the most common AMR gene, *tet(A)*, encoding an antibiotic efflux, shows resistance to at least 21 different antibiotics, including some of the critically important antibiotics shown in [23]. In addition to *tet(A),* eight other highly-occurring antimicrobial resistant genes were common in the studied countries, showing significant appearances in all but one or two countries—*aph(3″)-Ib*, *aph(6)-Id*, *blaEC*, *blaTEM-1*, *qacEdelta1*, *sul1*, *sul2*, and *aadA1.* All of the nine common highly occurring genes are found in *E. coli*, *Shigella*, and *Salmonella Enterica*. Our data revealed that *Salmonella enterica*, *E. coli*, and *Shigella* were the most common highly-occurring foodborne pathogens, showing significant appearances in most of the eight countries. This suggests that these pathogens may be prevalent because of their access to and expression of these AMR genes. According to the FDA, *Salmonella* and *E. coli* represent some of the most common causes of foodborne disease, and cause millions of illnesses every year [24].

### 4.3. Geographic and Trade Relations between AMR Genes of Different Countries

Of the eight countries studied, South Africa hosted the least number of common highly-occurring AMR genes. While all of the other countries had significant appearances of at least seven of the nine highly-occurring genes, South Africa had only two—*tet(A)* and *blaEC*. Rather, it had the most unique set of highly-occurring resistant genes with the least increase in AMR gene occurrence over time. Similarly, in both of the hierarchical clusters of the countries based on their foodborne pathogens and AMR genes (Figure 7 and Figure 8), South Africa was consistently separated from the other seven countries. This uniqueness may in part relate to a lack of access to technology and limited means for reporting gene occurrences. These limitations are further discussed in the section below, Limitations and Future Work. However, it may also be correlated to the limited involvement of South Africa in the global livestock market. Of the eight countries studied, South Africa has the lowest rate of importing and exporting of animal products and, thus, is the most isolated from world trade and the subsequent exchange of AMR genes and foodborne pathogens. South Africa, thus, had quite different foodborne pathogens and genes compared to the other seven countries. While similarly isolated geographically, Australia is one of the most developed countries in the world. Moreover, it is a key player in the global meat industry as the third largest exporter of beef and the 55th most populated country in the world [25]. Although it implements the most conservative antimicrobial policy, it exhibits trends in abundance and time of occurrence of AMR genes similar to the trends in other countries that are also heavily involved in the global meat industry. This suggests that the occurrence of AMR genes (and thus antimicrobial resistance) is most likely related to the involvement of a country in the global livestock trade market and its trade relations with other countries, rather than its geographic location or its antimicrobial policy. Another example of this relationship between a country and its trade patterns is the clustering of the US and the UK together in both Figure 7 and Figure 8. This may be associated with their close trade relations. For example, the US and the UK both underwent outbreaks of *Salmonella enterica* in recent years, specifically in 2013 and 2015 [26].

### 4.4. Limitations

The NPDIB database contains data from the last 30 years. During this time, this data has been affected by increasing technological accessibility; thus, an increase in the number of gene occurrences could possibly be attributed to a growing means of reporting it. In many countries, specifically developing ones, the lack of technology or uniform data collection policy may mean that less AMR genes and foodborne pathogens are reported. Moreover, since not every instance of every AMR gene was reported in each country, it is important to note that the database only contains a limited number of samples that may not be able to completely reflect the antimicrobial resistance status of each selected country. This work addressed these limitations by selecting the countries with the largest amounts of samples in various continents. It is hoped that economic and technological development can overcome these limitations and that more AMR samples can be obtained for countries all over the world in the future.

## 5. Conclusions

The NPDIB is a database containing genomic data for AMR genes and foodborne pathogen samples. The data has been provided from countries all over the world beginning in 2010. Data from this database during the period of 2010 to 2019 was analyzed to determine the occurrence of AMR genes over eight countries from various continents. Programs developed through the R-program performed principal component analysis and hierarchical clustering of high-dimensional data to identify genes and foodborne pathogens most involved in antimicrobial resistance. The results indicated that (1) *tet(A)*, *aph(3″)-Ib*, *aph(6)-Id*, *blaEC*, *blaTEM-1*, *qacEdelta1*, *sul1*, *sul2*, and *aadA1* were the 9 most common of 43 identified highly-occurring AMR genes; (2) *Salmonella enterica* and *E. coli* and *Shigella* were the most common highly-occurring foodborne pathogens; (3) chicken was the most prevalent meat carrier of AMR; and (4) South Africa had the most statistically unique resistance pattern. The large number of highly occurring AMR genes and the wide dissemination of many of these genes implies a potential rise of antimicrobial resistance. The spread of many highly occurring genes to numerous geographically distant countries with varying antimicrobial policies highlights the role of the global livestock trade in the transfer of AMR genes and foodborne pathogens. The identification of the nine globally significant highly-occurring genes, highly-occurring pathogens, and most prevalent meat carrier offer valuable insight for future research and development of new solutions for antimicrobial resistance.

## Figures and Tables

**Table 1:** Highest occurring AMR genes sorted and color coded by their presence in each country (indicated by PC and hierarchical clustering for countries: the US, the UK, South Africa, China, Australia, Canada, Mexico, and Brazil). The genes are listed from the highest occurring frequency (commonly present in all eight countries) to the lowest frequency (commonly present in only one country). Different occurring frequencies are marked in different colors, e.g., dark green for AMR genes commonly present in eight countries, and light green for AMR genes commonly present in seven countries.

All Genes	US	UK	South Africa	China	Australia	Canada	Mexico	Brazil
*tet(A)*	*tet(A)*	*tet(A)*	*tet(A)*	*tet(A)*	*tet(A)*	*tet(A)*	*tet(A)*	*tet(A)*
*aph(3″)-Ib*	*aph(3″)-Ib*	*aph(3″)-Ib*		*aph(3″)-Ib*	*aph(3″)-Ib*	*aph(3″)-Ib*	*aph(3″)-Ib*	*aph(3″)-Ib*
*aph(6)-Id*	*aph(6)-Id*	*aph(6)-Id*		*aph(6)-Id*	*aph(6)-Id*	*aph(6)-Id*	*aph(6)-Id*	*aph(6)-Id*
*blaEC*	*blaEC*	*blaEC*	*blaEC*	*blaEC*	*blaEC*	*blaEC*		*blaEC*
*blaTEM-1*	*blaTEM-1*	*blaTEM-1*		*blaTEM-1*	*blaTEM-1*	*blaTEM-1*	*blaTEM-1*	*blaTEM-1*
*qacEdelta1*	*qacEdelta1*	*qacEdelta1*		*qacEdelta1*	*qacEdelta1*	*qacEdelta1*	*qacEdelta1*	*qacEdelta1*
*sul1*	*sul1*	*sul1*		*sul1*	*sul1*	*sul1*	*sul1*	*sul1*
*sul2*	*sul2*	*sul2*		*sul2*	*sul2*	*sul2*	*sul2*	*sul2*
*aadA1*	*aadA1*	*aadA1*		*aadA1*	*aadA1*	*aadA1*		*aadA1*
*tet(B)*	*tet(B)*	*tet(B)*	*tet(B)*			*tet(B)*		*tet(B)*
*aadA2*		*aadA2*		*aadA2*	*aadA2*		*aadA2*	*aadA2*
*fosX*	*fosX*	*fosX*			*fosX*	*fosX*	*fosX*	
*lin*	*lin*	*lin*			*lin*	*lin*	*lin*	
*aadA*	*aadA*	*aadA*	*aadA*					*aadA*
*floR*	*floR*			*floR*			*floR*	*floR*
*fosA7*	*fosA7*	*fosA7*					*fosA7*	*fosA7*
*aph(3′)-Ia*	*aph(3′)-Ia*			*aph(3′)-Ia*	*aph(3′)-Ia*			
*dfrA12*				*dfrA12*	*dfrA12*		*dfrA12*	
*blaCMY-2*	*blaCMY-2*	*blaCMY-2*						*blaCMY-2*
*bcrB*	*bcrB*					*bcrB*		
*bcrC*	*bcrC*					*bcrC*		
*abc-f*		*abc-f*				*abc-f*		
*sul3*				*sul3*	*sul3*			
*qnrB19*							*qnrB19*	*qnrB19*
*blaCTX-M-55*				*blaCTX-M-55*				
*blaOXA-193*	*blaOXA-193*							
*catA1*			*catA1*					
*mph(A)*				*mph(A)*				
*tet(O)*	*tet(O)*							
*aadA5*				*aadA5*				
*ble*				*ble*				
*bleO*				*bleO*				
*fosA3*				*fosA3*				
*mcr-1.1*				*mcr-1.1*				
*oqxA*				*oqxA*				
*oqxB*				*oqxB*				
*aac(2′)-Ic*			*aac(2′)-Ic*					
*blaA*			*blaA*					
*erm(37)*			*erm(37)*					
*cmlA1*					*cmlA1*			
*dfrA5*					*dfrA5*			
*qacL*					*qacL*			
*fosA*							*fosA*	

**Table 2:** AMR genes with an increasing occurrence from 2010 to 2019 in the eight countries

US	UK	South Africa	China	Australia	Canada	Mexico	Brazil
*aadA1*	*aadA1*	*aph(3″)* *-Ib*	*aadA2*	*blaTEM-1*	*aadA1*	*aadA2*	*aph(3″)* *-Ib*
*blaOXA-193*	*aadA2*	*aph(6)-Id*	*blaTEM-1*	*dfrA5*	*tet(B)*	*blaTEM-1*	*aph(6)-Id*
*blaTEM-1*	*abc.f*	*blaEC*	*ble*	*sul2*		*dfrA12*	*blaCMY-2*
*floR*	*aph(3″)* *-Ib*	*blaTEM-1*	*floR*			*fosA*	*blaTEM-1*
*qacEdelta1*	*aph(6)-Id*	*dfrA5*	*mph(A)*			*qacEdelta1*	*floR*
*sul1*	*blaCMY.2*	*sul2*	*oqxA*			*sul1*	*fosA7*
*tet(A)*	*blaTEM.1*	*tet(A)*	*oqxB*			*sul2*	*sul2*
*tet(O)*	*fosA7*	*tet(B)*	*qacEdelta1*			*tet(A)*	*tet(A)*
	*qacEdelta1*		*sul1*				*tet(B)*
	*sul1*		*sul2*				
	*sul2*						
	*tet(A)*						

**Table 3:** Highest occurring AMR pathogens sorted and color coded by their presence in each country (the US, the UK, South Africa, China, Australia, Canada, Mexico, and Brazil). Note: red indicates AMR pathogen presence in 7–8 out of the 8 countries; orange indicates AMR pathogen presence in 2–5 out of the 8 countries; and yellow indicates the AMR pathogen presence in 1 out of the 8 countries.

	Countries	US	UK	South Africa	China	Australia	Canada	Mexico	Brazil
Pathogens									
*E. coli and Shigella*		x	x	x	x	x	x	x	x
*Salmonella enterica*		x	x	x	x		x	x	x
*Listeria monocytogenes*		x	x			x	x	x	
*Klebsiella pneumoniae*					x				x
*Mycobacterium tuberculosis*				x					
*Campylobacter jejuni*		x							
*Pseudomonas aeruginosa*									x
*Enterobacter*					x				
*Cronobacter*					x				

**Table 4:** Foodborne pathogens with an increasing occurrence in the eight selected countries

US	UK	South Africa	China	Australia	Canada	Mexico	Brazil
*Campylobacter jejuni*	*Listeria monocytogenes*	*E. coli* and *Shigella*	*Klebsiella pneumoniae*	*E. coli* and *Shigella*	*E. coli* and *Shigella*	*E. coli* and *Shigella*	*Salmonella enterica*
	*Salmonella enterica*		*Salmonella enterica*				

**Table 5:** Ranking of beef, chicken, pork, and turkey based on their prevalence in each country. The ranking is on a scale of 1 to 4, 1 represents the most prevalent carrier while 4 represents the least prevalent carrier.

	Countries	US	UK	South Africa	China	Australia	Canada	Mexico	Brazil	Total Rank
Meats										
Beef		2	4	No Data	4	1	1	1	4	17
Chicken		1	1	No Data	1	4	2	2	1	12
Pork		4	2	No Data	2	4	4	4	2	22
Turkey		4	4	No Data	4	4	4	4	4	28

## Supplementary Materials

The following are available online at https://www.mdpi.com/1660-4601/17/2/472/s1, Table S1: Resistance Mechanisms and Drug Class of Highly-occurring AMR genes.
