# Supplementary material for: Investigation of Incidents and Trends of Antimicrobial Resistance in Foodborne Pathogens in Eight Countries from Historical Sample Data
Source: Int J Environ Res Public Health. 2020 Jan 10;17(2):472. doi: 10.3390/ijerph17020472 (PMC7014410; doi:10.3390/ijerph17020472)
Supplement: Supplementary file 1 [file ijerph-17-00472-s001.pdf]

International Journal of
Environmental Research
and Public Health

MDPI

Supplementary Document

Investigation of Incidents and Trends of Antimicrobial Resistance in Foodborne Pathogens in Eight Countries from Historical Sample Data

Katherine Yang [1], Annie Wang [1], Matthew Fu [1], Aaron Wang [1], Kevin Chen [1], Qian Jia [2], Zuyi (Jacky) Huang [1, *]

Supplementary Table s1. Resistance Mechanisms and Drug Class of Highly-occurring AMR genes.

Genes	Drug	Resistance Mechanism
aac(2')-Ic	aminoglycoside antibiotic	antibiotic inactivation (acetylation of the aminoglycoside antibiotic on the amino group at position 2')
aadA	aminoglycoside antibiotic	antibiotic inactivation
aadA1	aminoglycoside antibiotic	antibiotic inactivation
aadA2	aminoglycoside antibiotic	antibiotic inactivation
aadA5	aminoglycoside antibiotic	antibiotic inactivation
abc.f	macrolide antibiotic, lincosamide antibiotic, streptogramin antibiotic, tetracycline antibiotic, oxazolidinone antibiotic, phenicol antibiotic, pleuromutilin antibiotic)	antibiotic target protection
aph(3)-Ia	aminoglycoside antibiotic	antibiotic inactivation
aph(3)-Ib	aminoglycoside antibiotic	antibiotic inactivation
aph(6)-Id	aminoglycoside antibiotic	antibiotic inactivation
bcrB	peptide antibiotic, peuromutilin antibiotic, cephalisporin, penam, fluorquinolone antibiotic, acridine dye, nitroimidazole, rifamycin antibiotic, tetracycline antibioic, macrolide antibiotic	antibiotic efflux (efflux pump complex or subunit conferring antibiotic resistance)
bcrC	peptide antibiotic	antibiotic target alteration
blaA	cephalosporin, penam	antibiotic inactivation
blaCMY.2	cephalosporin, penam	antibiotic inactivation
blaCTX-M-55	cephalosporin, penam	antibiotic inactivation
blaEC	cephalosporin, penam	antibiotic inactivation
blaOXA-193	cephalosporin, penam	antibiotic inactivation
blaTEM.1	cephalosporin, penam	antibiotic inactivation
ble	glycopeptide antibiotic	antibiotic inactivation
bleO	glycopeptide antibiotic	antibiotic inactivation
catA1	phenicol antibiotic	antibiotic inactivation
cmlA1	tetracycline antibiotic, antibacterial free fatty acids, acridine dye, rhodamine, isoniazid, lincosamide antibiotic, fluoroquinolone antibiotic, penam, cephalosporin, glycylcycline, fosfomycin, macrolide antibiotic, bicyclomycin, rifamycin antibiotic, phenicol antibiotic, diaminopyrimidine antibiotic, peptide antibiotic, oxazolidinone antibiotic, benzalkonium chloride, nucleoside antibiotic, nitroimidazole antibiotic	antibiotic afflux
dfrA12	diaminopyrimidine antibiotic	antibiotic target replacement
dfrA5	diaminopyrimidine antibiotic	antibiotic target replacement
erm(37)	macrolide antibiotic, lincosamide antibiotic, streptogramin antibiotic	antibiotic target alteration
floR	tetracycline antibiotic, phenicol antibiotic, diaminopyrimidine antibiotic, fosfomycin, cephalosporin, acridine dye, rhodamine, nitroimidazole antibiotic, benzalkonium chloride, nucleoside antibiotic, antibacterial free fatty acids, oxazolidinone antibiotic, rifamycin antibiotic, peptide antibiotic, glycylcycline, macrolide antibiotic, bicyclomycin, penam, isoniazid, lincosamide antibiotic, fluoroquinolone antibiotic	major facilitator superfamily (MFS) antibiotic efflux pump
fosA3	fosfomycin	antibiotic inactivation
fosA7	fosfomycin	antibiotic inactivation Ibreaks the epoxide ring of the molecule)
fosx	fosfomycin	antibiotic inactivation (ribosomal protection)
lin	lincosamide antibiotic	antibiotic inactivation
mcr.1.1.	peptide antibiotic	antibiotic target alteration

(continued)

mph(A)	macrolide antibiotic	antibiotic inactivation
oqxA	tetracycline antibiotic, acridine dye, triclosan, antibacterial free fatty acids, nitrofuran antibiotic, aminoglycoside antibiotic, phenicol antibiotic, diaminopyrimidine antibiotic, aminocoumarin antibiotic, monobactam, glycylcycline, macrolide antibiotic, penam, carbapenem, fluoroquinolone antibiotic	antibiotic efflux (efflux pump complex or subunit conferring antibiotic resistance)
oqxB	tetracycline antibiotic, glycylcycline, macrolide antibiotic, nitrofuran antibiotic, aminoglycoside antibiotic, antibacterial free fatty acids, triclosan, fluoroquinolone antibiotic, acridine dye, penam, carbapenem, phenicol antibiotic, diaminopyrimidine antibiotic, aminocoumarin antibiotic, monobactam	antibiotic efflux
qacEdelta1	quaternary ammonium compounds	antibiotic efflux
qacL	quaternary ammonium compounds	antibiotic efflux
qnrB19	fluoroquinolone antibiotic	antibiotic target protection
sul1	sulfonamide antibiotic, sulfone, antibiotic	antibiotic target replacement
sul2	sulfonamide antibiotic, sulfone, antibiotic	antibiotic target replacement
sul3	sulfonamide antibiotic, sulfone antibiotic	antibiotic target replacement
tet.A.	tetracycline antibiotic, benzalkonium chloride, nucleoside antibiotic, oxazolidinone antibiotic, rifamycin antibiotic, phenicol antibiotic, diaminopyrimidine antibiotic, peptide antibiotic, glycylcycline, fosfomycin, macrolide antibiotic, bicyclomycin, penam, cephalosporin, acridine dye, rhodamine, isoniazid, lincosamide antibiotic, fluoroquinolone antibiotic, nitroimidazole antibiotic, antibacterial free fatty acids	antibiotic efflux
tet.B.	tetracycline antibiotic, antibacterial free fatty acids, rifamycin antibiotic, phenicol antibiotic, diaminopyrimidine antibiotic, peptide antibiotic, glycylcycline, fosfomycin, macrolide antibiotic, bicyclomycin, penam, cephalosporin, acridine dye, rhodamine, isoniazid, lincosamide antibiotic, fluoroquinolone antibiotic, nitroimidazole antibiotic, benzalkonium chloride, nucleoside antibiotic, oxazolidinone antibiotic	antibiotic efflux
tet(O)	tetracycline antibiotic	antibiotic target protection
